# Soret Effect on MHD Casson Fluid over an Accelerated Plate with the Help of Constant Proportional Caputo Fractional Derivative

**DOI:** 10.1021/acsomega.3c07311

**Published:** 2024-02-23

**Authors:** Shajar Abbas, Mushtaq Ahmad, Mudassar Nazar, Zubair Ahmad, Muhammad Amjad, Hakim AL Garalleh, Ahmed Zubair Jan

**Affiliations:** † Centre for Advanced Studies in Pure and Applied Mathematics, Bahauddin Zakariya University Multan 60000, Pakistan; ‡ Department of Mathematics, Institute of Southern Punjab Multan 66000, Pakistan; § Applied College, Mahala Campus, King Khalid University, P.O. Box 9004, Abha 61413, Saudi Arabia; ∥ Center of Bee Research and Its Products, King Khalid University, P.O. Box 9004, Abha 61413, Saudi Arabia; ⊥ Department of Mathematics, Comsats University Islamabad, Vehari Campus, Vehari 61100, Pakistan; # Department of Mathematical Science, College of Engineering, University of Business and Technology-Dahban, Jeddah 21361, Saudi Arabia; ¶ Faculty of Mechanical Engineering, Wroclaw University of Science and Technology, Wroclaw 50-370, Poland

## Abstract

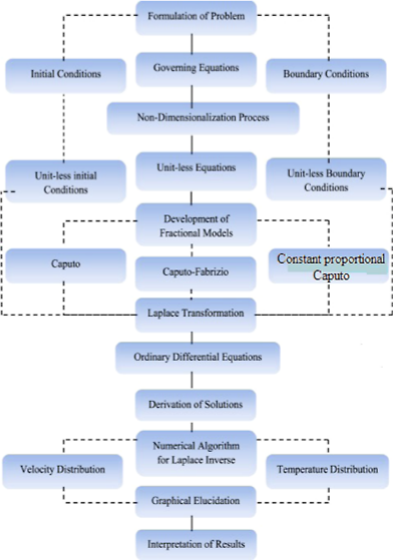

Non-Newtonian fluid flow is significant in engineering and biomedical applications such as thermal exchangers, electrical cooling mechanisms, nuclear reactor cooling, drug delivery, blood flow analysis, and tissue engineering. The Caputo operator has emerged as a prevalent tool in fractional calculus, garnering widespread recognition. This research aims to introduce a novel derivative by merging the proportional and Caputo operators, resulting in the fractional operator known as the constant proportional Caputo. In order to demonstrate this newly defined operator’s dynamic qualities, it was employed in the analysis of the unsteady Casson flow model. In addition, the current work shows an analytical analysis to determine the Soret effect on the fractionalized MHD Casson fluid over an oscillating vertical plate. Fractional partial differential equations (PDEs) are used to formulate the problem along with IBCs. The introduction of appropriate nondimensional variables converts the PDEs into dimensionless form. The precise solutions to the fractional governing PDEs are then determined by the Laplace transform method. Velocity, concentration, and temperature profiles; the impacts of the Prandtl number; fractional parameter β and γ; and Soret and Schmidt numbers are graphically depicted. The profiles of temperature, concentration, and velocity rise with rising time and fractional parameters. Interestingly, as the Casson flow parameter is higher, fluid velocity decreases closest to the plate but increases away from the plate. Tables showing the findings for the skin-friction coefficient, Sherwood, and Nusselt numbers for a range of flow-controlling parameter values are provided. Furthermore, an investigation is undertaken to compare fractionalized and ordinary velocity fields. The results suggest that the fractional model employing a constant proportional derivative exhibits a quicker decay than the model incorporating conventional Caputo and Caputo-Fabrizio operators.

## 1 Introduction

Heat and mass transfer processes hold immense significance from an industrial perspective, captivating the attention of numerous researchers and scientists. In the realm of modern technologies and diverse industrial applications, the theory of non-Newtonian fluids exerts a profound influence due to the limitations of Newtonian fluid models in capturing a wide range of flow characteristics. Non-Newtonian fluids exhibit complex relationships involving shear strain rate and stress, transcending the simplistic assumptions of Newtonian fluid models. The theory of non-Newtonian fluids finds significant application in contemporary engineering, namely, within the petroleum sector, where it plays a crucial role in extracting crude oil from various petroleum reservoirs. In contrast to Newtonian fluids, whose properties often prove inadequate, non-Newtonian fluids demand the development of more sophisticated models to accurately represent their intricate behavior. The significance of non-Newtonian fluids has grown exponentially in recent decades, particularly within the research community. These fluids boast a vast array of ever-expanding applications in various industrial sectors, including large-scale heating and cooling systems, plastic extrusion, polymer processing, oil pipeline friction reduction, well drilling, fluid friction minimization, biological materials, flow tracing, plastic foam processing, biomedical flow analysis, food processing industries, lubrication processes, emulsions, chemical processing, slurries, and mud handling.

Numerous scientists have dedicated their efforts to studying non-Newtonian fluids, considering a variety of fluid geometries. For this reason, advancement and improved quality of life are greatly aided by the modeling and simulation of non-Newtonian fluid flow processes. Researchers have investigated a diverse range of non-Newtonian fluid models, each with its own unique computational properties. For instance, while the power-law model effectively captures viscosity characteristics, it fails to account for the effects of elasticity. This motivates researchers and mathematicians to delve deeper into the study of these complex fluids. For theoretical research as well as real-world applications in contemporary engineering, a methodical examination of these fluid flow models is crucial.^1−4^

There have been several rheological models proposed in order to comprehend the characteristics of flow and heat transmission. Casson flow model^5^ is one of them. This model does not satisfy Newton’s law of viscosity which was created by Casson (1995). Since Casson fluid’s characteristics relate to the shear stress relation, it is classified as a non-Newtonian fluid. Blood, tomato juices, soup, and juice are a few examples of Casson fluids. Inextricably linked to this model are some freeze flows. Yield stress may be seen in this model. Dash et al.^6^ investigated how yield pressure affects the motion of a Casson flow in a comparable permeable medium confined in a tube. Fluid with homogeneous and heterogonous reactions is investigated in refs (7–10).

Shashikumar et al.^11^ was taken into consideration in order to analyze the effects of Casson nanofluids nonlinear flow among plates that are held side by side. Hayat et al.^12^ studied non-Newtonian Casson flow with magnetohydrodynamics with the impact of Dufour and Soret. This model, along with a variety of flow characteristics and combinations, has been employed by several academics^13−15^ to achieve a variety of objectives. Fractional models have a few drawbacks since the single Kernel encountered several issues throughout the modeling process. In addition to extending fractional integrals, Caputo and Riemann–Liouville developed the idea of singular Kernel-based fractional derivative operators.

New fractional operators have been introduced in order to address this issue like the fractional derivative of Prabhakar, Caputo–Fabrizio, Atangana–Baleanu, and some others.^16,17^ Baleanu et al.^18^ worked with the fractional operator constant proportional Caputo (CPC); the Riemann–Liouville integral and Caputo fractional derivative are combined to produce this. Yavuz et al.^19^ offered the precise solution and investigation of an operator for a Caputo fractional specified a second grade fractionalized fluid. Caputo and Fabrizio^20^ discovered novel fractional derivatives enhanced by various scientists to solve practical issues. There are some important references for fractional calculus.^21−24^

Numerous other scientific disciplines, including mathematics, physics, geophysics, biology, etc., have also adopted the usage of fractional order derivatives. These areas are examined in refs (25–28). Tamoor et al.^29^ studied the stretching cylinder to look at how the MHD affects the flow of the Casson fluid. Using the stationary motion of a Newtonian flow, Nadeem et al.^30^ examined the influence of an MHD on a curvilinear surface. A time-dependent micropolar fluid and a curved sheet were used in the investigation. Saleh et al.^31^ investigated the impact of injection or suction. Today’s food companies criticize the Casson flow model. Casson’s flowing model was used by the cocoa and chocolate manufacturing sectors to show how chocolate behaves rheologically. Additionally, in modern times, the rheological model for human blood is distributed using the Casson model. Casson fluid flows over various geometries in a variety of conditions are mentioned in refs (32–34).

Researchers Damseh et al.^35^ investigated coupled mass and heat transfer via free convection of a micropolar, viscous, heat-generating or -absorbing fluid flow near a continuously moving vertical porous infinitely long surface in the presence of a first-order chemical reaction. For unsteady coupled mass and heat transfer via mixed convection flow over a vertical cone rotating in an ambient liquid with a time-dependent angular velocity, Chamkha and Rashad^36^ investigated the Soret and Dufour effects in the presence of a MHD and chemical reaction. Takhar et al.^37^ discuss the MHD flow across a moving plate in a fluid that rotates with free stream velocity, Hall effects, and a magnetic field.

Chamkha and Khaled^38^ studied the issue of simultaneous mass and heat transfer by free convection from a semi-infinite inclined plate when an external magnetic field is present and internal heat production or absorption effects are described. The influence of thermophoresis and heat generation or absorption was explored in the Chamkha and Issa^39^ study of heat and mass transport across a semi-infinite, permeable flat surface in the setting of continuous, two-dimensional, laminar, hydromagnetic flow. Chamkha and Khaled^40^ showed hydromagnetic coupled mass and heat transfer aided by free convective from a porous surface housed in a fluid-saturated porous medium. Kumar et al.^41^ explored an electrically conducting, incompressible, and viscous liquid for mixed convective boundary layer flow on a vertical plate amid thermal radiation and an induced magnetic field. In Chamkha,^42^ coupled heat and mass transport in the presence of electromagnetic radiation and a magnetic field are emphasized together with free convective boundary layer flow across a permeable isothermal truncated cone. Some useful discoveries may be found in refs (43–48).

In the current study, an exact approach is applied to analyze the constant wall concentration and temperature for an unsteady Casson fluid boundary layer flow across a vertically accelerating plate. The set of partial differential equations (PDEs) is analytically solved utilizing the Laplace method. The influence of several dimensionless quantities on concentration, velocity, and heat is thoroughly investigated. Subsequently, Stehfest’s and Tzou’s algorithms are used to invert the transformed results. The solutions obtained for the problem are graphically presented, and the influence of relevant parameters is elucidated using graphs. The influence of the Soret effect is investigated by varying parameters in the governing equations. The concentration, temperature, and velocity profiles obtained using the fractional derivative exhibit a more pronounced decay compared to those obtained with the ordinary derivative. This study is innovative in that it applies the new fractional hybrid operator, the CPC fractional derivative,^18^ and expands on the work^49^ for the Casson flow. As far as we are aware, no such conclusion has been achieved for the Casson fluid generated by the Soret effect with CPC fractional derivatives. According to this, the fractional derivative is a more suitable choice for achieving controlled velocity, mass, and heat profiles. The present work holds significant potential for various applications, including the design of geothermal systems, electronic materials, and solar energy systems.

## 2 Mathematical Formulation

**Figure 1:**
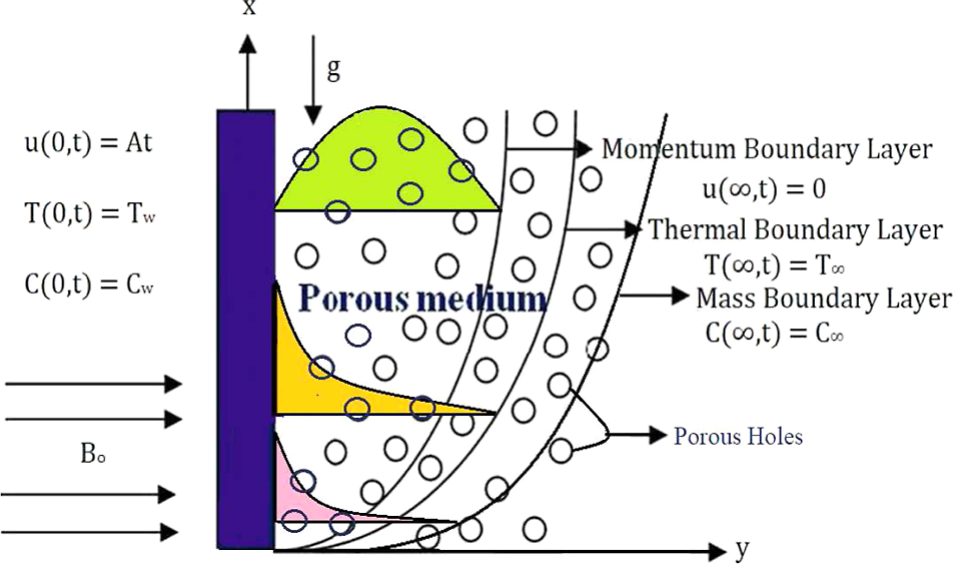
Geometry of flow and coordinate system.

The flow of a Casson flow that is incompressible over an infinitely accelerated vertical plate is examined here. Flow is contained inside the region of *y* > 0, where *y* is a perpendicularly measured coordinate to the plate. At initial rest, both the liquid and plate have a constant temperature of *T*_∞_ at *t* = 0. A speed *At*, where *A* denotes the oscillation plate, causes the plate to begin accelerating in its plane at time *t* > 0. The plate’s temperature rises simultaneously to *T*_w_ and is then kept constant. Temperature and velocity are dependent on time and space variables *y* and *t*, as shown in Figure 1. Following are the forms of the momentum and heat equations when considering unidirectional flow and Boussinesq’s approximation^49,50^

Momentum equation
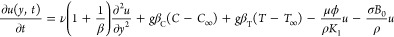
1

Energy equation
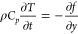
2the generalized Fourier law
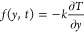
3

Concentration equation with thermo-diffusion
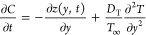
4the generalized Fick’s law
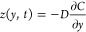
5with declaration of field variables at the boundary
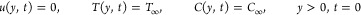
6
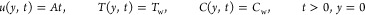
7
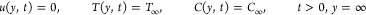
8where the fluid’s velocity is *u*, fluid temperature is *T*, *C* is the fluid concentration, the density of fluid is ρ, the dynamic viscosity is μ, the coefficient of thermal expansion β_T_, and the mass expansion coefficient β_C_, electrical conductivity is σ, the constant pressure specific heat at a fixed pressure is *C*_*p*_, and the identical strength of magnetic field *B*_0_, *k* is the heat conductivity, while chemical molecular diffusivity is *D*.

The modified typical Ohm’s law for the greater magnitude magnetic field is expressed as follows^51−54^
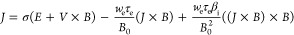
9

Equation 9 is constituent parts are
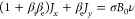
10
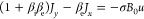
11

On solving eqs 10 and 11, we get
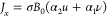
12
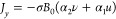
13the ion slip and Hall effects are neglected in the governing equation because according to our flow supposition, this effect is very minute so it cannot be considered.

By inserting the dimensionless variables given below
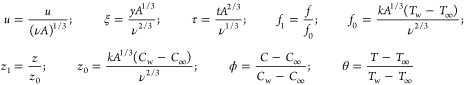
14into eqs 1–8, we have
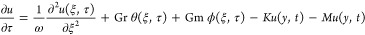
15
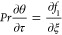
16
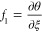
17
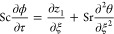
18
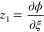
19where *Sr* is Soret effect, *Gm* is mass Grashof number, *Gr* is Grashof number, *Sc* is the Schmidt number, *M* is the magnetic parameter, and *Pr* is the Prandtl number. The nondimensional IBCs is
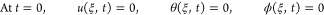
20
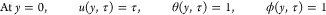
21
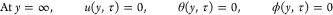
22

## 3 Fractional Model with CPC Fractional Differential Operator

We will create a fractional modeling of the physical issue in this part. The definition and examples of the novel fractional derivative are found in ref (18)
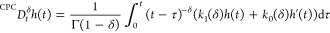
23

CPC’s Laplace transform is presented in ref (18)
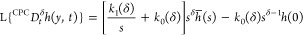
24

We obtain the fractional PDEs
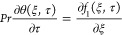
25
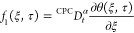
26
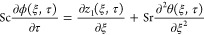
27
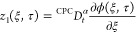
28

Substituting eq 26 in eq 25 and eq 28 in eq 27, we get
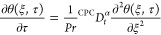
29
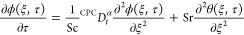
30

The fractional PDEs presented in eqs 29 and 30 are effectively solved using the Laplace technique, a robust technique employed to obtain analytical solutions for initial value problems.

### 3.1 Solution of Temperature Equation

With the use of the Laplace transform technique, we will solve energy eq 29 with boundary constraints (20)_2_–(21)_2_ in this section.
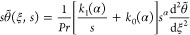
31

Assuming the following IBCs
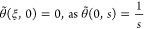
32

Result of eq 31 subject to eq 32, we have
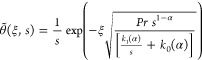
33

Equation 33 has an exponential expression, and the square root term is complex, hence it is impossible to obtain its direct inverse Laplace transform. To analytically obtain the required result, we thus write it in an alternative form as
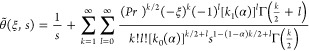
34invert Laplace transform to eq 34 gives
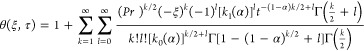
35which is the final solution for the temperature field.

### 3.2 Solution of Concentration Equation

Apply Laplace method on eq 30, we get
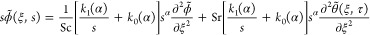
36

Rearrange the above equation
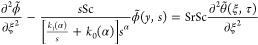
37with conditions
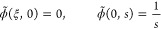
38

The concentration field solution to eq 37 satisfies conditions (38), we have
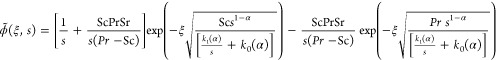
39

In summation notation, eq 39 is represented as
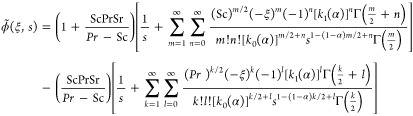
40

Inverse Laplace transformation of above eq 40 results in
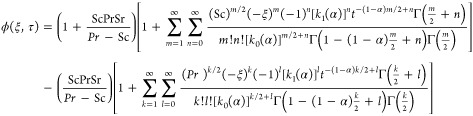
41

### 3.3 Solution of Velocity Equation

Using CPC fractional derivative and utilizing the Laplace method to eq 15 with constraints (20)_1_ and (21)_1_, we obtain
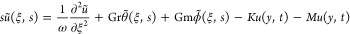
42

Putting values of (ξ,s) and θ̃(ξ,s) in eq 42, we get
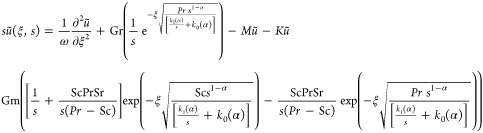
43

Rearrange the above equation
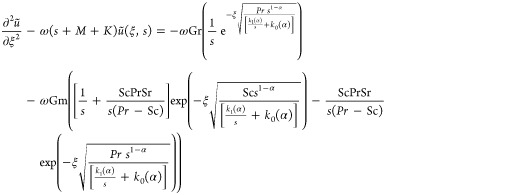
44satisfy
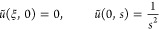
45

We obtain the velocity profile’s using eq 45 in eq 44
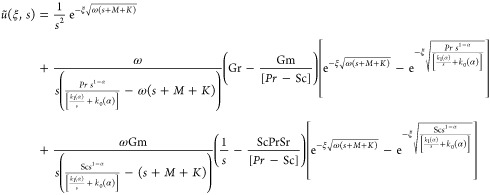
46

Using Zakian’s procedure
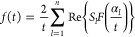
47for the inverse Laplace transform form of eq 46. Final calculation for the velocity field is then obtained. Inverse Laplace transform by Zakian’s method is described in refs (55 and 56).

## 4 Results and Discussion

The Soret effect in the magnetohydrodynamics flow of Casson fractional flow over an accelerated infinite vertical plate in the current study, considering generalized mass and heat transfer through a permeable media. The study presents semianalytical findings for the velocity fields and exact findings for the mass and heat. Additionally, the physical impact of the relevant parameters is illustrated through graphical representations of the heat, mass, and velocity fields.

**Figure 2:**
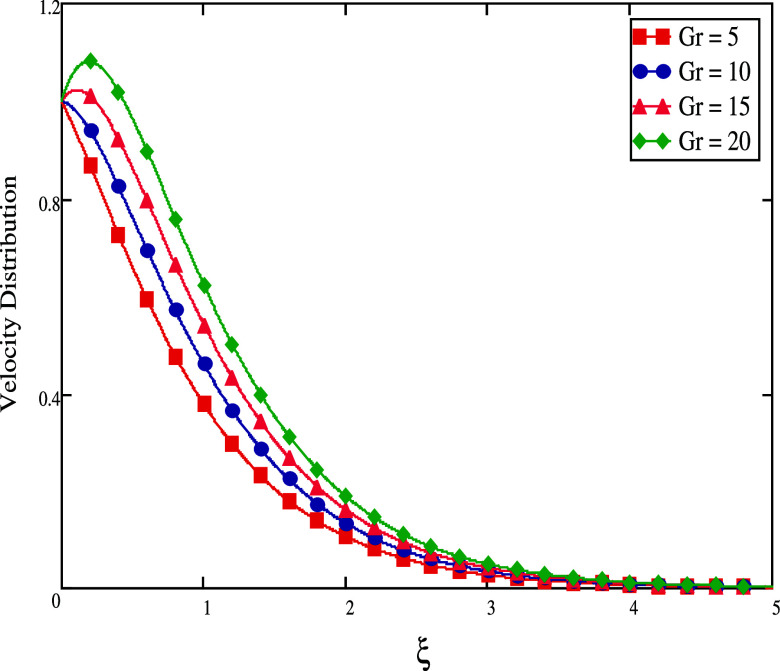
For different *Gr* values, velocity profiles.

**Figure 3:**
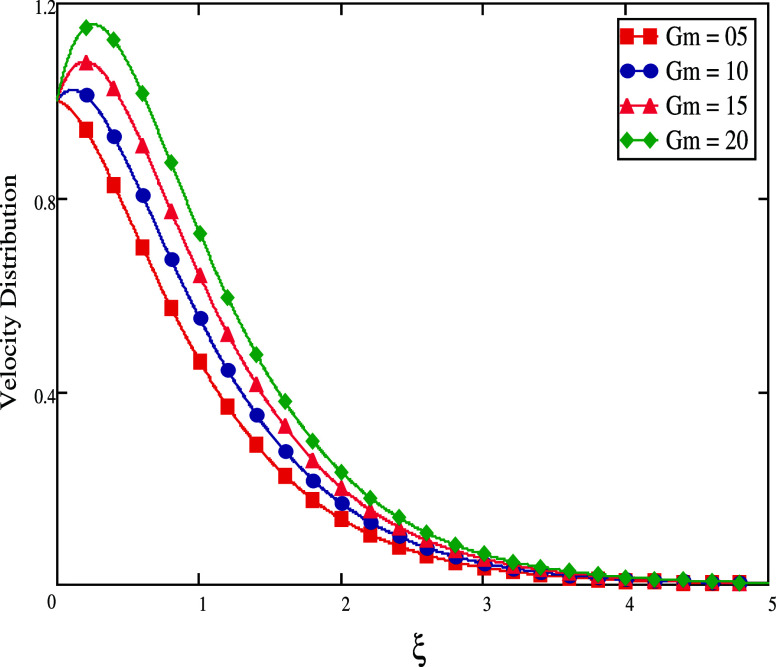
For different *Gm* values, velocity profiles.

**Figure 4:**
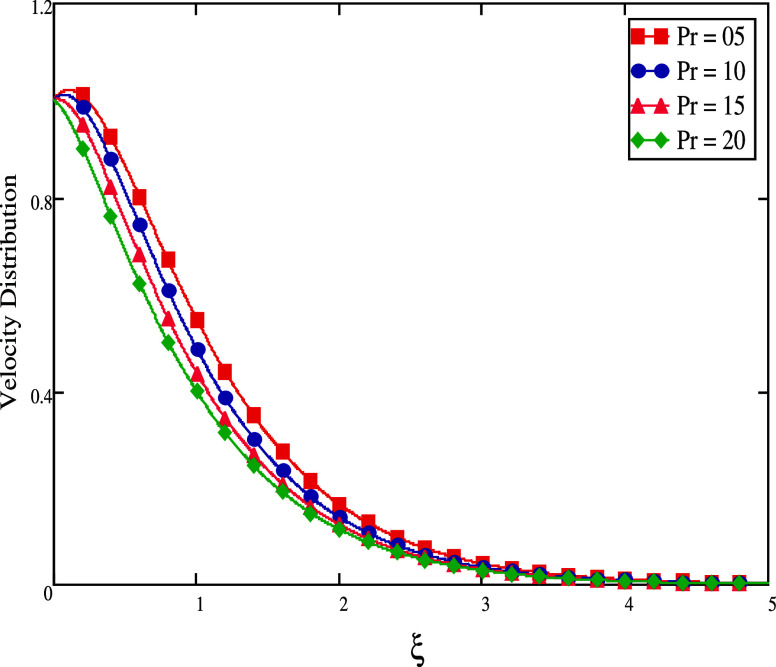
For different *Pr* values, velocity profiles.

**Figure 5:**
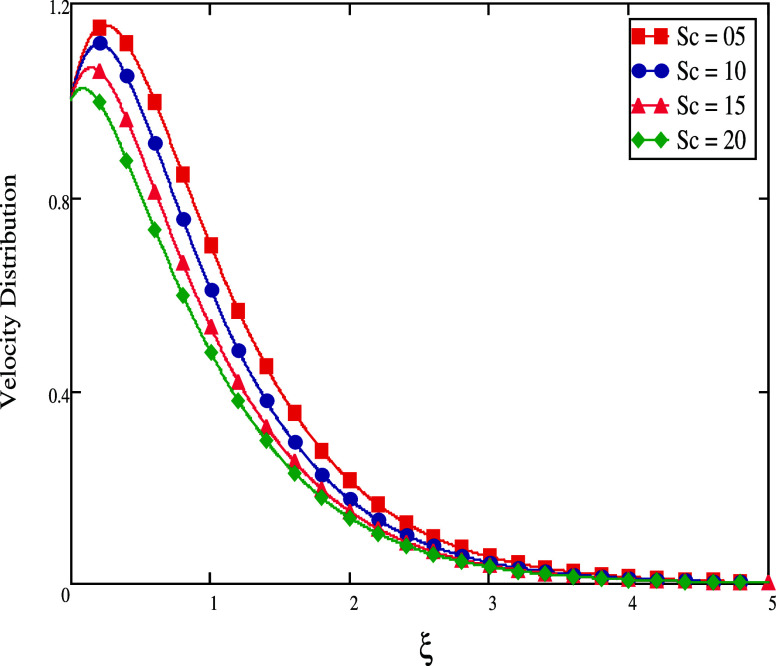
For different *Sc* values, velocity profiles.

Figure 2 illustrates the impacts of the Grashof number *Gr*. As we raise the value of *Gr*, velocity fluid rises. *Gr* measures the relationship between the heat buoyant force to the viscous force. When *Gr* = 0, no convection flow exists. If *Gr* is more than zero, the plate is chilled outside; if *Gr* is less than zero, the plate is heated externally. Grashof number controls the flow regime in natural convection. *Gm* effects on the velocity profile is depicted in Figure 3. Velocity is understood to be quicker with higher *Gm* levels. The speed of the velocity increases because *Gm* is connected to buoyancy forces, which increase natural convection. In Figure 4, a drop in fluid velocity is evident along with an increase in the *Pr* (Prandtl number). The temperature gradient would be reduced by increasing the fluid’s heat diffusivity by rising the *Pr* value. Therefore, the loss of thermal kinetic energy causes a drop in velocity fluid. When the Schmidt number, *Sc*, varies, Figure 5 depicts the fluid’s behavior. This figure shows that there is a correlation between a rise in the Schmidt number and a fall in fluid velocity. The Schmidt number is the ratio between kinematic viscosity and molecular diffusion. Molecular diffusion tends to decrease as the Schmidt number increases, which slows down fluid movement.

**Figure 6:**
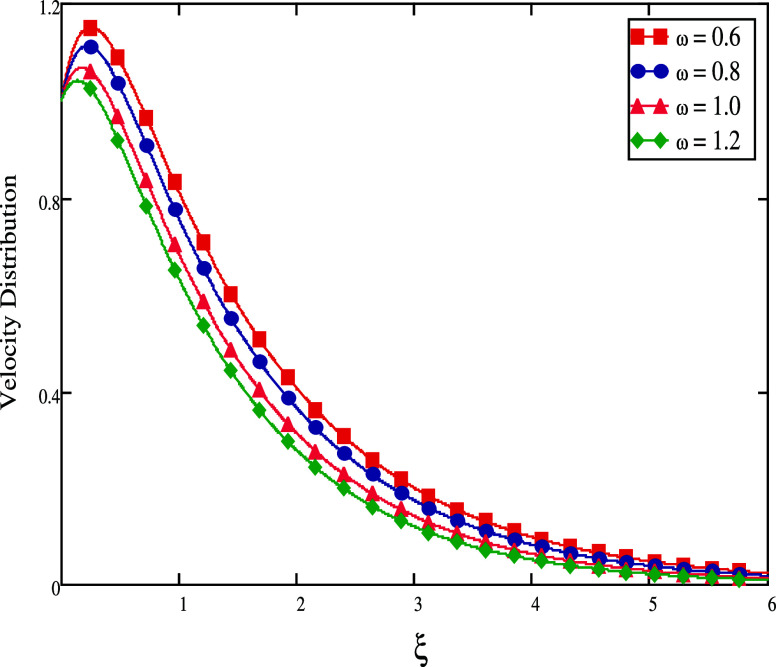
For different ω values, velocity profiles.

**Figure 7:**
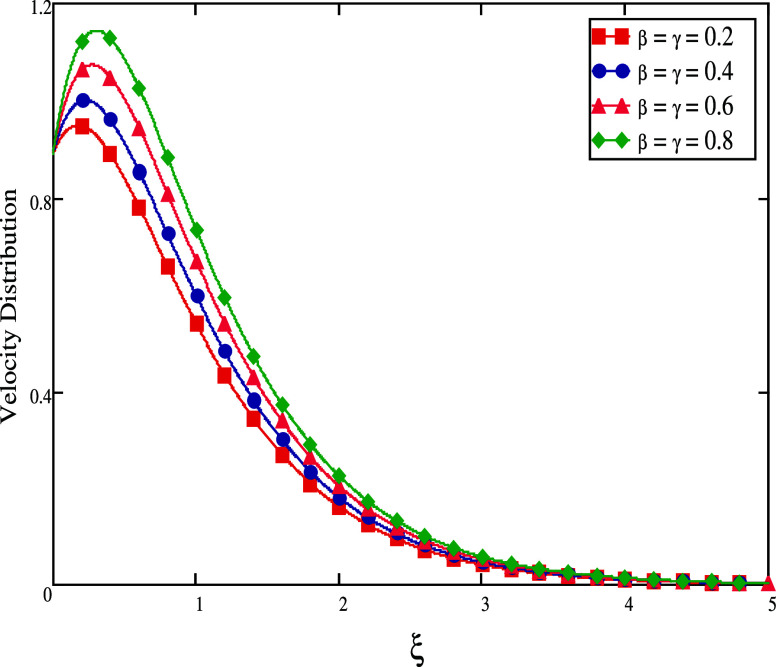
Effect of distinct fractional values for large time.

**Figure 8:**
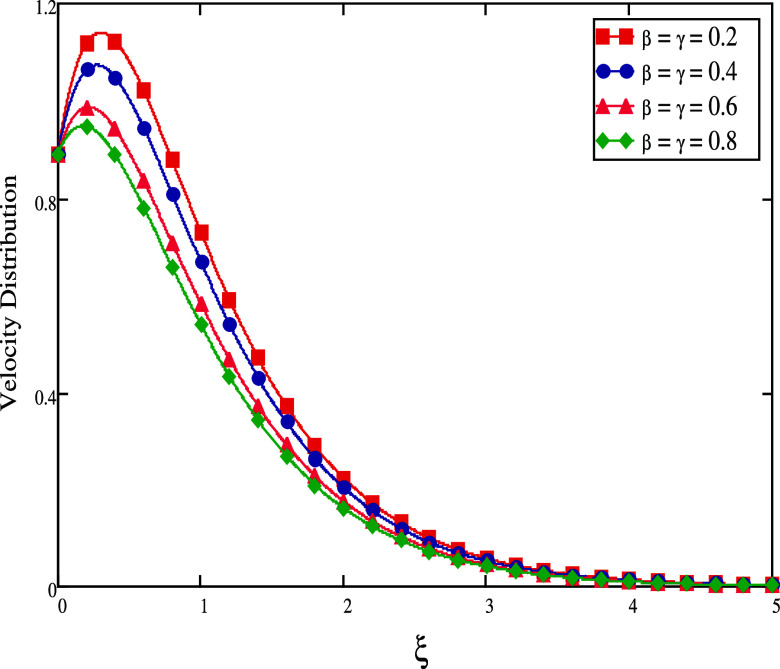
Effect of distinct fractional values for small time.

**Figure 9:**
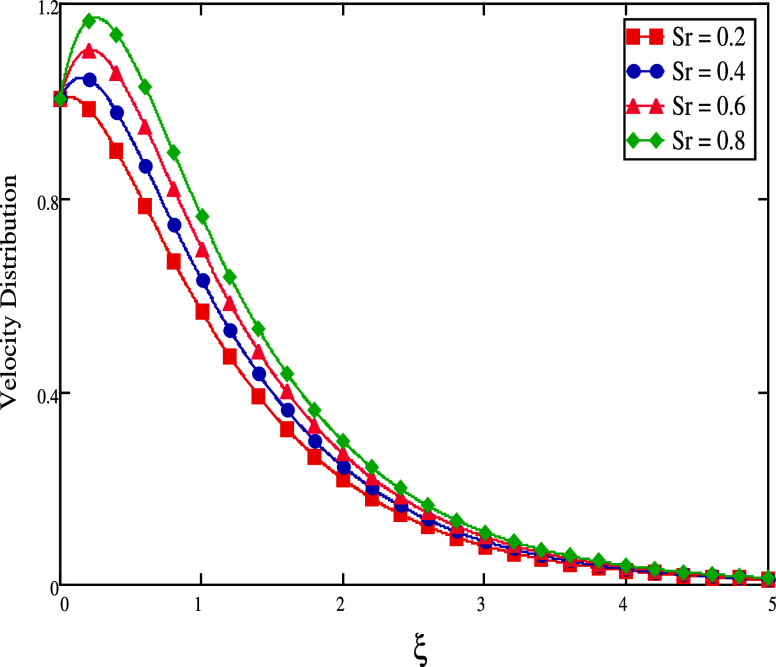
For different Soret values, velocity profiles.

Figure 6 shows how ω affects the velocity field. Increasing ω causes the drag forces to become firmer, which tends to diminish the velocity field, which causes the value of the velocity fluid to decay. Figures 7 and 8 show the influence of velocity fractional parameter profiles for small and large time durations. As we increase the fractional parameter values for a short time period, as shown in Figure 7, the fluid velocity decreases. The velocity rises for large time durations, as seen in Figure 8. This may be explained physically since increasing the β and γ increases the thermal and momentum boundary layers, which in turn rises the velocity distribution for a significant amount of time. The thermo-diffusion or Soret effect (*Sr*) over the velocity field is discussed in Figure 9. Because of the rising fluctuations in the *Sr*, an augmenting flow pattern is seen. In the flow domain, the concentration gradient is influenced by the temperature gradient, adding to the mass flux. As a result, when the *Sr* value increases, there is a corresponding increase in the mass flux, which causes the flow current to rise and the flow speed to increase accordingly.

**Figure 10:**
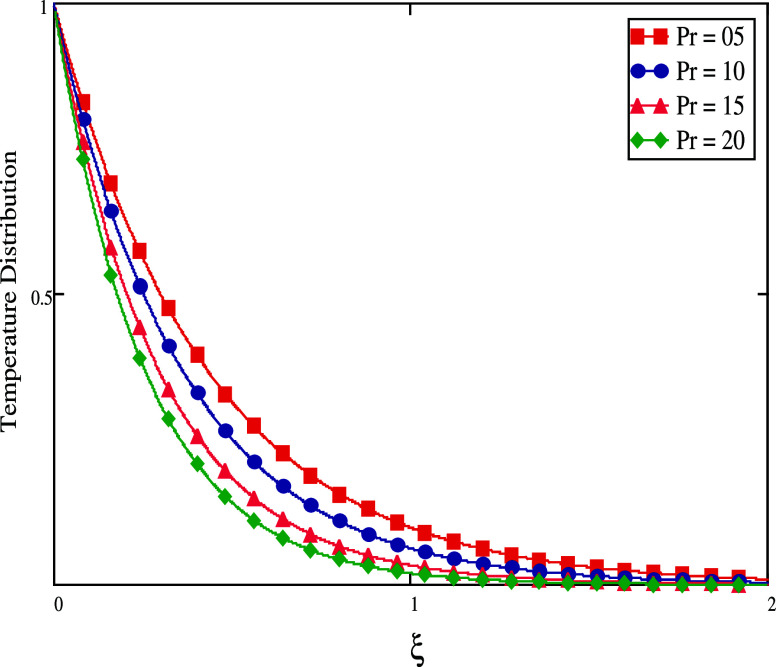
Effect of distinct fractional values for small time.

**Figure 11:**
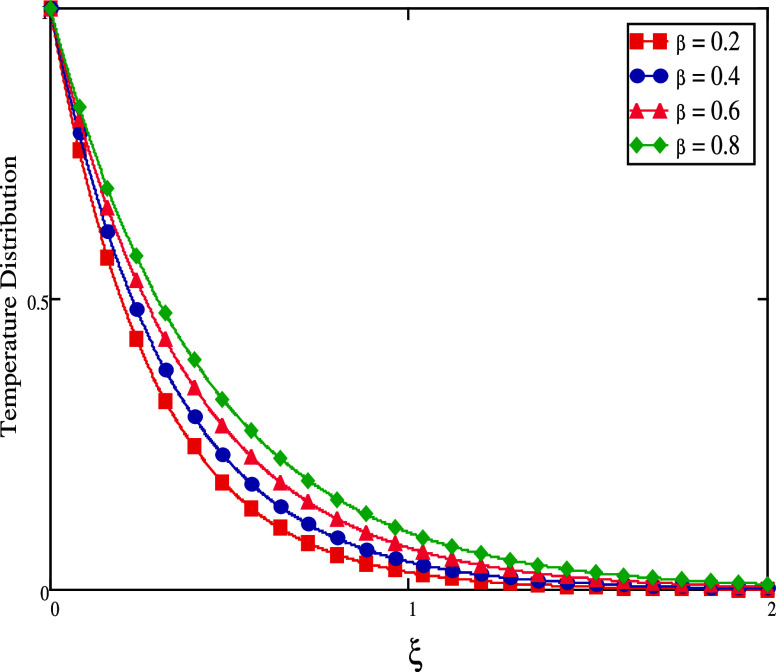
Temperature profiles for large time.

**Figure 12:**
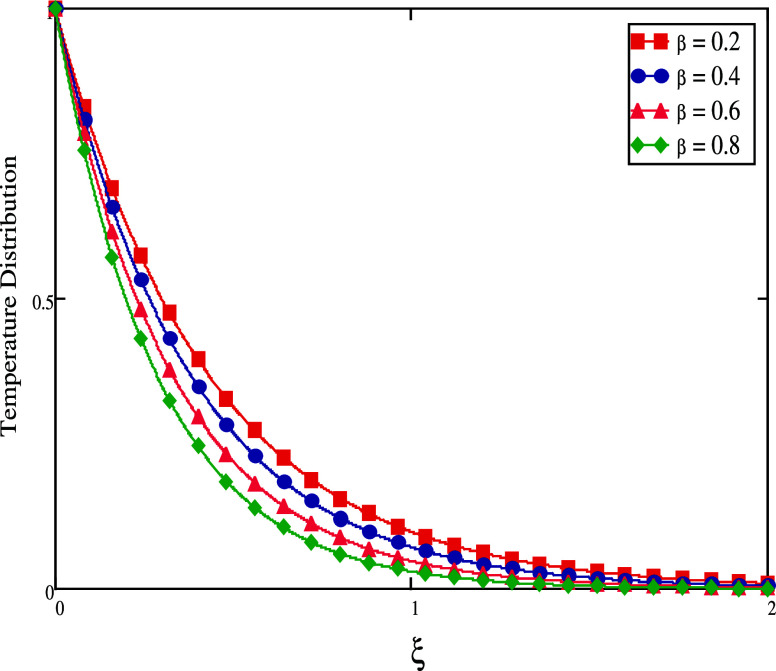
Temperature profiles for small time.

The *Pr* is used to calculate the thickness of the thermal boundary layer. The drop in heat diffusion rate is brought on by the increase in *Pr* which dominates momentum diffusivity over fluid movement. As a consequence, *Pr* increases, thermal boundary layer thickness diminishes, and heat profile becomes lower, as seen in Figure 10. Figures 11 and 12 show, for both short and long times, the impact of β on temperature profiles. When time is short, the fluid temperature drops as the γ values are increased (see Figure 11). The temperature rises as the amount of time increases (see Figure 12).

**Figure 13:**
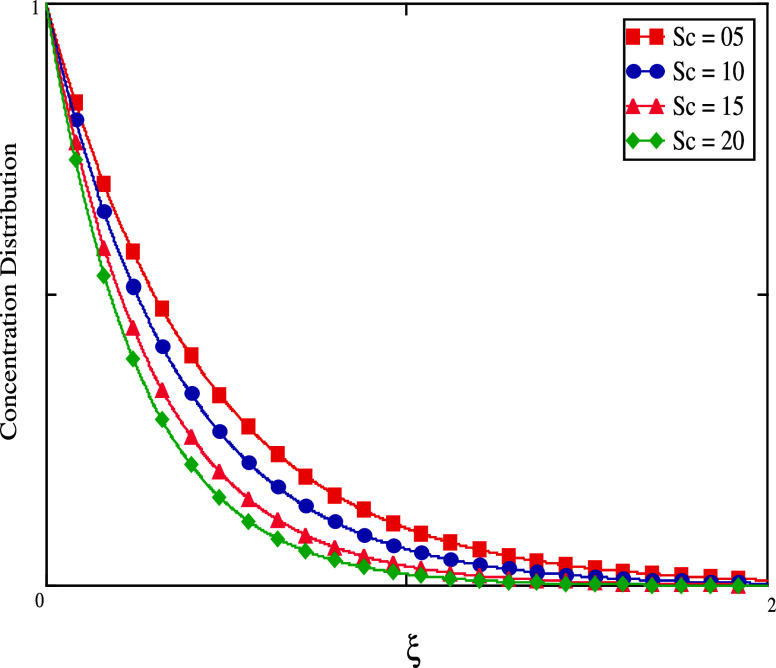
Concentration profiles for distinct *Sc* values.

**Figure 14:**
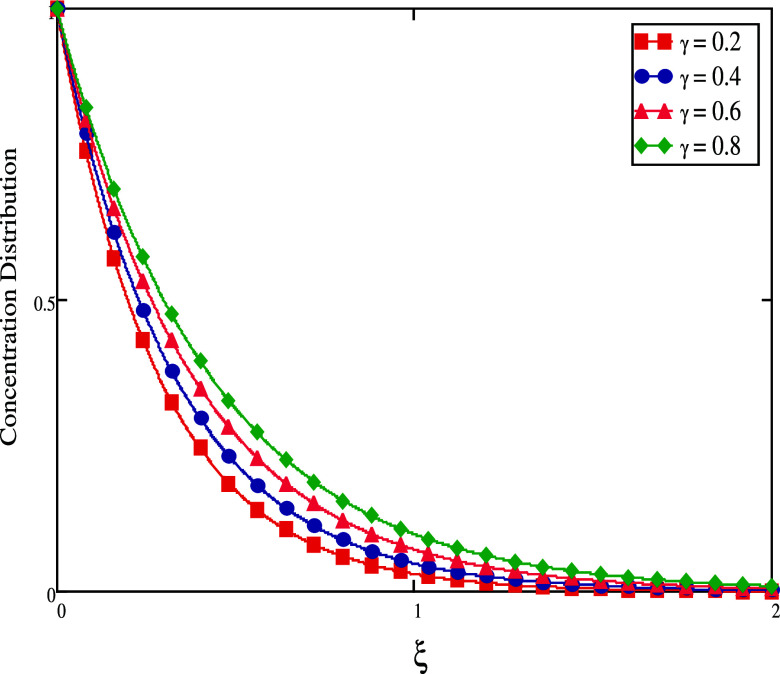
Concentration profiles for large time.

**Figure 15:**
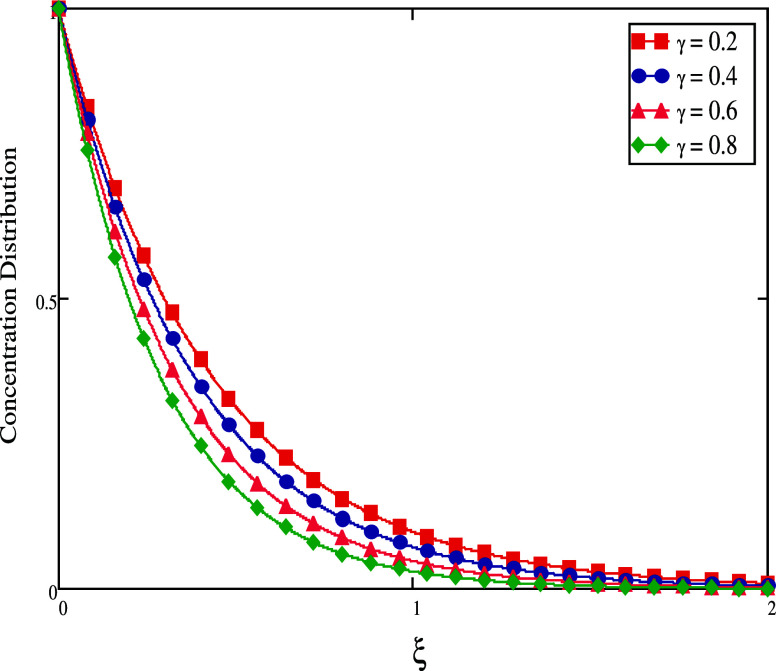
Concentration profiles for small time.

**Figure 16:**
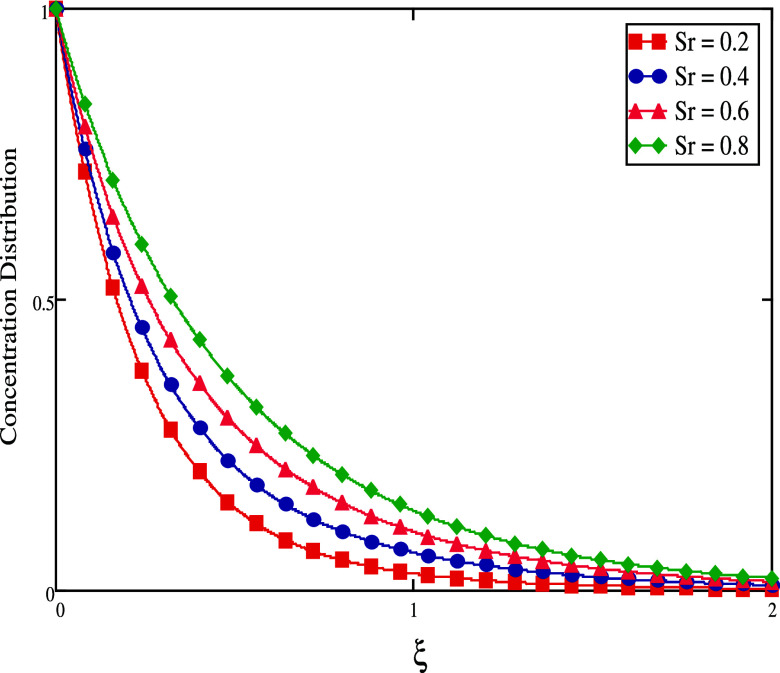
Concentration profiles for distinct *Sr* values.

In Figure 13, it is intended to investigate how the Schmidt number has an impact on concentration. By keeping the Schmidt number’s value constant while varying the other factors, it has been discovered that for larger Schmidt number values, accordingly, the field variable concentration cannot be increased. Since this is the situation, increasing the *Sc* values increases the viscous force that affects fluid flow, hence lowering the concentration flow. Figures 14 and 15 show the impact of γ on mass profiles when time has both small and large values. When time is short, we increase γ values, and the fluid concentration decreases (see Figure 14). When time is big, the concentration rises (see Figure 15). The graphical behavior of Sr on temperature is shown in Figure 16. This figure shows that temperature rises by raising the values of Sr.

**Figure 17:**
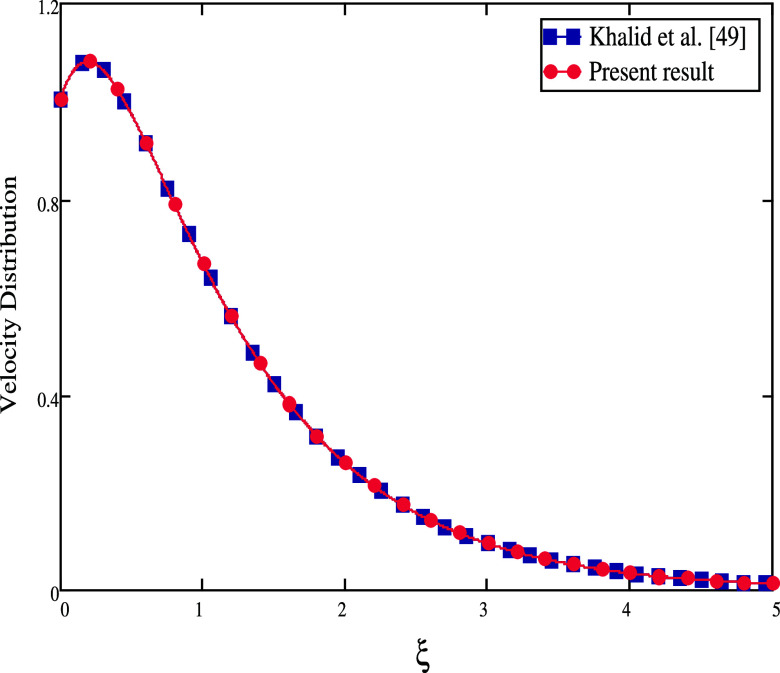
Velocity distribution for comparison of our work with Khalid et al.^49^ as α = 1, *k*_1_ = 0, *k*_0_ = 1, *Gm* = 0, *K* = 0, *M* = 0, and *Sr* = 0.

**Figure 18:**
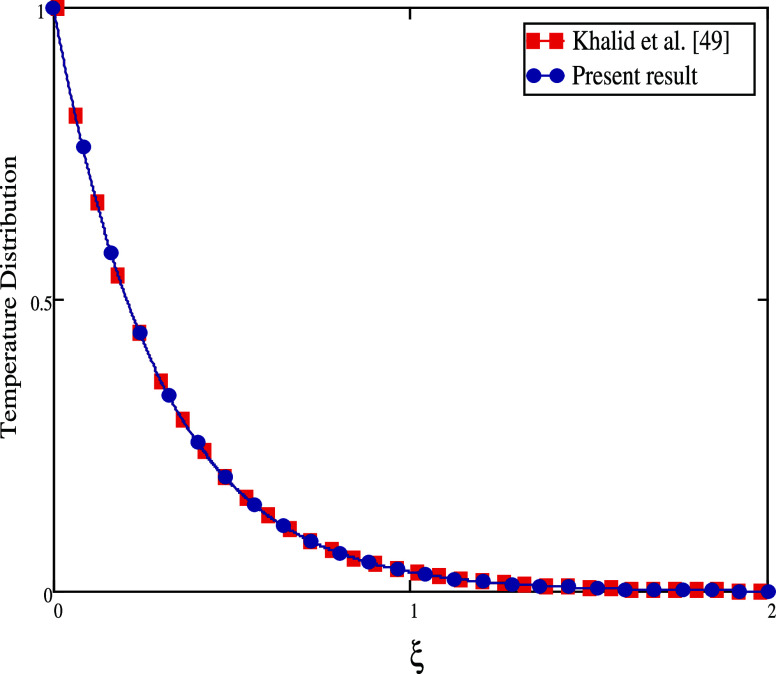
Temperature distribution for comparison of our work with Khalid et al.^49^ as α = 1, *k*_1_ = 0, *k*_0_ = 1, *Gm* = 0, and *Sr* = 0.

**Figure 19:**
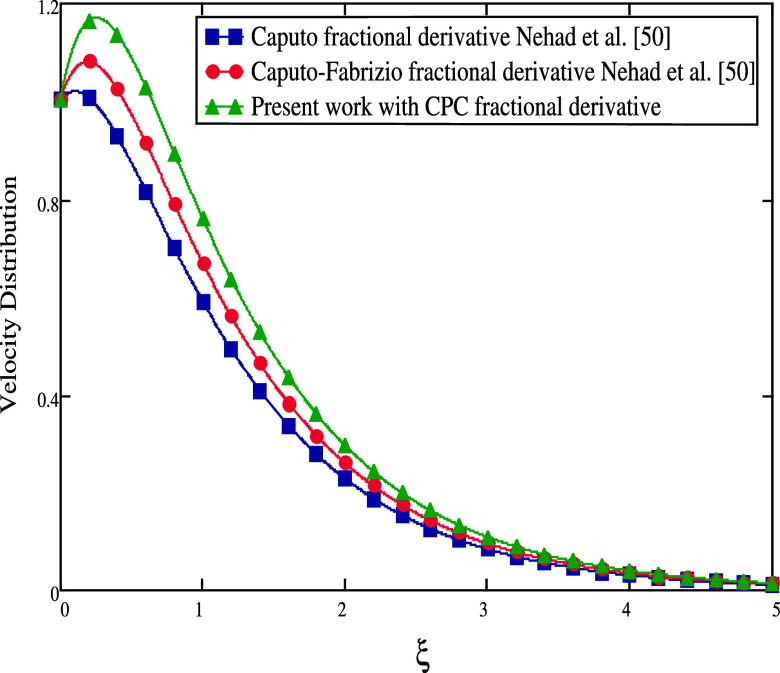
Comparisons between different fractional derivatives α = 0.5.

**Figure 20:**
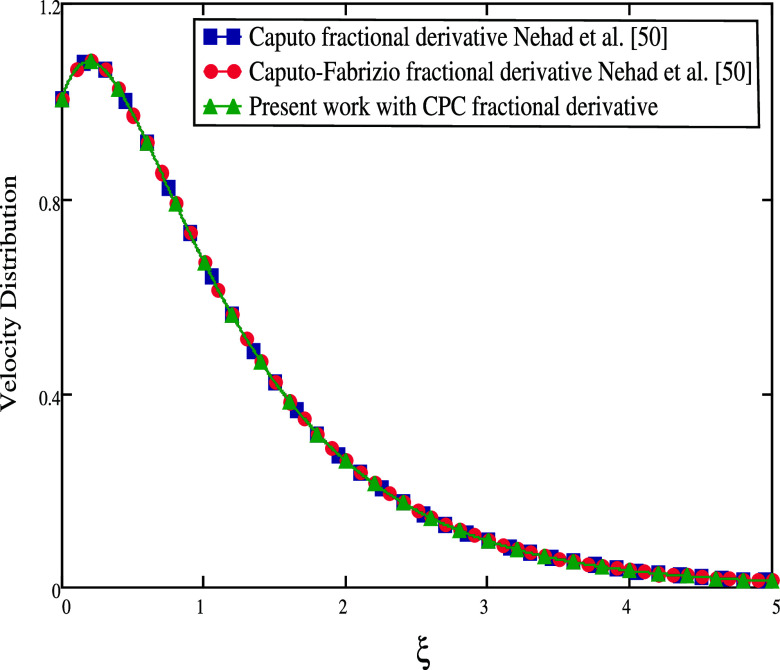
Comparisons between different fractional derivatives α → 1.

The comparison of the velocity and temperature distribution between the current study and Khalid et al.^49^ is shown in Figures 17 and 18, respectively. If we take fractional parameters, α = 1, *k*_1_ = 0, *k*_*o*_ = 1, *Gm* = 0, *K* = 0, *M* = 0, and *Sr* = 0 in Khalid et al.,^33^ the fact that the velocity profiles are identical demonstrates the validity of the current work. Additionally, Figure 19 compares the results of the current study with other fractional operators, Caputo and Caputo–Fabrizio, utilized in Nehad et al.^50^ in the absence of β = ∞, *Gm* = 0, *K* = 0, *M* = 0, and *Sr* = 0. The fluid profiles are the same as seen in Figure 20 if α is set to 1. A Casson fluid with a CPC fractional derivative is the best option, according to the figures, to improve fluid motion.

By raising the fractional parameter values, as shown in Tables 1–3, skin friction, heat, and mass transfer rates can all be improved.

**Table 1:** Skin Friction

β, γ	*t* = 1	*t* = 2	*t* = 3	*t* = 4
0.1	0.071	0.097	0.116	0.132
0.2	0.076	0.103	0.124	0.141
0.3	0.078	0.106	0.128	0.146
0.4	0.080	0.109	0.132	0.151
0.5	0.083	0.113	0.136	0.166
0.6	0.085	0.117	0.141	0.161
0.7	0.088	0.120	0.145	0.166
0.8	0.091	0.124	0.150	0.172
0.9	0.094	0.128	0.155	0.177
1.0	0.097	0.132	0.160	0.182

**Table 2:** Nusselt Number

β	*t* = 1	*t* = 2	*t* = 3	*t* = 4
0.1	1.151	1.177	1.196	1.212
0.2	1.156	1.183	1.204	1.221
0.3	1.158	1.186	1.208	1.226
0.4	1.160	1.189	1.212	1.231
0.5	1.163	1.193	1.216	1.236
0.6	1.165	1.197	1.221	1.241
0.7	1.168	1.200	1.225	1.246
0.8	1.171	1.204	1.230	1.252
0.9	1.174	1.208	1.235	1.257
1.0	1.177	1.212	1.240	1.262

**Table 3:** Sherwood Number

γ	*t* = 1	*t* = 2	*t* = 3	*t* = 4
0.1	0.067	0.095	0.114	0.131
0.2	0.073	0.102	0.123	0.142
0.3	0.076	0.106	0.128	0.147
0.4	0.079	0.110	0.133	0.152
0.5	0.082	0.115	0.138	0.158
0.6	0.085	0.119	0.143	0.164
0.7	0.089	0.124	0.149	0.170
0.8	0.092	0.128	0.154	0.177
0.9	0.096	0.133	0.160	0.183
1.0	0.100	0.138	0.166	0.189

## 5 Skin Friction

The non-dimensional skin friction is given in eq 48
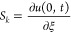
48

## 6 Nusselt Number

According to the Nusselt number, the rate of heat transport is provided by eq 49
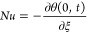
49

## 7 Sherwood Number

Equation 50 provides the mass transfer rate expressed in terms of the Sherwood number.
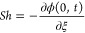
50

*Gr*, *Gm*, *Pr*, *Sc*, and *M* are among the variable parameters whose numerical values are used in the study.

The *Gr* is a nondimensional quantity that is utilized to describe spontaneous or free convection flow. The particular application determines the typical values for *Gr*. It can vary between 10^4^ and 10^12^ for flows of vertical plates. It can be anywhere between 10^8^ and 10^12^ for horizontal plate flows. It may differ considerably more for enclosures and other geometries. The Gm is a nondimensional quantity that describes natural convection induced by buoyancy forces arising from density variations induced by mass gradients. The *Gm* value range is comparable to that of the *Gr* for free convection. In typical applications, Gm can span from 10,000 to 10^12^. A dimensionless quantity known as the Prandtl number (*Pr*) indicates the ratio of heat diffusivity to the diffusivity of momentum. The *Pr* typical values vary depending on the fluid under consideration. It is around 0.7 for air, about 7 for water, and anything from 10 to 100 for oils. A dimensionless quantity called the Schmidt number (*Sc*) is used to quantify how momentum and mass diffusivity interact in a fluid. The fluid under consideration affects the typical values for *Sc* as well. This equals 600 for water and about 0.7 for air. The magnetic number (*M*), a dimensionless quantity, is used to describe how a magnetic field affects a fluid’s flow. The magnetic field’s strength and the fluid’s properties determine the typical values for *M*. It is possible for it to be between 10^–6^ and 10^–3^ for light magnetic fields and between 1 and 10 for strong magnetic fields.

Table 4 below lists the ranges of the various parameters that were employed in this investigation.^57−59^

**Table 4:** Ranges of the Various Parameters Employed in This Investigation

name of parameters	ranges
*Gm*	3.50–7.20
*Gr*	2.50–5.60
*M*	1.50–3.50
*Pr*	2.20–4.20
*Sc*	2.10–4.30

## 8 Conclusions

This work employs precise results for an unsteady Casson fluid boundary layer flows over a vertical plate that is oscillating and has constant wall heat and mass. The Laplace transform is used to solve the nondimensional governing equations. Measured values of temperature, concentration, and velocity are shown graphically. The following parameter includes the Casson parameter, Prandtl number, Grashof number, Schmidt number, Soret number, mass Grashof number, and fractional parameter as well as their impacts on velocity, concentration, and temperature.

- The results of this study can be used to design more efficient heat exchangers and chemical reactors.
- When *Gr*, *Gm*, β, and γ are increased, velocity rises; however, when *Pr*, *Sc*, and ω are increased, velocity falls.
- Temperature rises when time and fractional parameters are increased but decreases as *Pr* is raised.
- Concentration rises when time and fractional parameters are increased but decreases as *Sc* is raised.
- In biomedical engineering, the Soret effect in Casson fluids can be relevant for drug delivery systems. By exploiting the concentration gradients established by the Soret effect, controlled release of drugs or therapeutic agents can be achieved in targeted regions. The non-Newtonian behavior of the Casson fluid can further influence the flow behavior and enhance the efficiency of drug delivery.
- In the oil and gas industry, the Soret effect in Casson fluids can be significant in enhanced oil recovery (EOR) techniques. EOR methods aim to improve the extraction of oil from reservoirs, and the Soret effect in Casson fluids can help enhance the displacement of oil by injecting fluids with specific temperature profiles. This can lead to improved oil recovery rates and increased production efficiency.
- The Soret effect in Casson fluids can also impact heat transfer systems. By manipulating the temperature gradient, concentration gradients can be established, which can modify the rate at which heat is transferred through the fluid. This effect can be utilized in applications such as cooling systems, heat exchangers, and thermal management devices to improve thermal transfer efficiency.
- In the food sector, the Soret effect in Casson fluids can find uses in processes such as thermal sterilization and pasteurization. By leveraging the concentration gradients established by the Soret effect, heat can be distributed more effectively within the fluid, leading to improved heat treatment processes and preservation of food products.

## Data Availability

No data associated in the manuscript.
